# Efficacy and safety of fermented *Prunus mume* vinegar on fatigue improvement in adults with unexplained fatigue: A randomized controlled trial

**DOI:** 10.3389/fnut.2022.990418

**Published:** 2022-11-09

**Authors:** Jung In Choi, Ye Li Lee, Sang Yeoup Lee

**Affiliations:** ^1^Family Medicine Clinic and Biomedical Research Institute, Pusan National University Yangsan Hospital, Yangsan, South Korea; ^2^Integrated Research Institute for Natural Ingredients and Functional Foods, Yangsan, South Korea; ^3^Department of Medical Education, Pusan National University School of Medicine, Yangsan, South Korea

**Keywords:** fermentation, *Prunus mume* vinegar, fatigue, dietary supplements, polysaccharides, randomized controlled trial (MeSH)

## Abstract

**Background:**

The accumulation of fatigue leads to reduced physical, emotional, psychological, and social functions.

**Objectives:**

Fermented *Prunus mume* vinegar (PV) improves fatigue in animals; however, studies in humans have not been conducted. We aimed to examine the effects and safety of consuming fermented PV for 8 weeks on fatigue indices in adults with unexplained fatigue while considering the placebo effect.

**Methods:**

A randomized, double-blind, placebo-controlled trial was conducted in adults of >19 years, who were diagnosed with unexplained fatigue for at least 1 month. Eighty participants were randomly assigned to receive daily 70 mL of fermented PV (2.56 mg/g, chlorogenic acid, and 15.3 mg/g, citric acid) or a placebo for 8 weeks. At baseline and 4 and 8 weeks after treatment, the participants were visited for blood tests (liver enzyme, glucose, creatinine, lactate, malondialdehyde [MDA], and creatine kinase [CK]) and questionnaires (Fatigue Severity Scale [FSS], fatigue visual analog scale [VAS], Beck Depression Inventory [BDI], the Korean version of the Brief Encounter Psychosocial Instrument [BEPSI-K], EQ-5D-3L, and EQ-VAS]).

**Results:**

Fermented PV supplementation for 8 weeks did not remarkably improve the fatigue indices when compared to placebo. Additionally, differences in fatigue VAS, BDI, BEPSI-K, EQ-5D-3L, EQ-VAS, lactate, CK, and MDA concentrations between the groups were not observed. However, FSS had positively correlated with fatigue VAS, BDI, and BEPSI-K, whereas it was negatively correlated with EQ-5D-3L and EQ-VAS at the baseline and 8 weeks. None of the participants reported adverse events.

**Conclusion:**

The efficacy of fermented PV did not exceed the efficacy of placebo in adults with unexplained fatigue.

**Clinical trial registration:**

[ClinicalTrials.gov], identifier [NCT04319692].

## Introduction

Fatigue can be defined as a state of inadequate energy to perform activities. People occasionally experience temporary fatigue when they are overworked or overtired. Fatigue is also related to various factors, such as mental, physical, and social factors. Temporary fatigue can be relieved by identifying the cause and resolving it. However, some people experience persistent long-term fatigue without any particular reason. Persistent unrelenting fatigue leads to decreased resistance, worsening of disease, difficulty concentrating, and memory problems, thereby lowering the individual’s work and productivity. It also affects emotional and psychological wellbeing (1). Thus, fatigue is a significant problem that affects the performance of daily activities (2). Additionally, fatigue is associated with medical conditions (such as chronic liver disease (3), cancer (2), stroke (4), rheumatic diseases, chronic kidney disease, type 1 diabetes mellitus, Parkinson’s disease, and multiple sclerosis (5), health behavior (such as smoking (5), alcohol consumption and physical inactivity (6), mental illness (such as depression and anxiety (5), work-related strains, or even workplace safety issues such as occupational accidents (7).

Generally, sufficient rest and adequate nutrition are required for the improvement of fatigue. Moreover, if necessary, the underlying disease should be managed. Additionally, some supplements may help relieve fatigue. Previous randomized controlled trials have shown that acetyl-L-carnitine supplementation in patients with hepatitis C virus infection (8), aminolevulinic acid supplementation in those with subjective constant physical fatigue (9), coenzyme Q10 supplementation in patients with juvenile fibromyalgia (10) or multiple sclerosis (11), and Q10 plus nicotinamide adenine dinucleotide supplementation in patients with chronic fatigue syndrome (12) have had positive effects on fatigue improvement.

*Prunus mume* is the fruit of the plum tree, and its fruit extract has been used for cooking and medicinal purposes in East Asia since ancient times (13). Presently, Asians mainly use them for the treatment of symptoms, such as gastrointestinal dysfunction, stomach/liver protection, and fatigue recovery. They can be processed and used in several products, including syrups, liquors, sauces, and juice. *Prunus mume* vinegar (PV) is consumed as a traditional beverage (14) and is rich in organic acids, including citric acid (15). Its extract reduces lactate dehydrogenase activity and increases citrate synthase activity in the skeletal muscles immediately after exercise loading in rats (16). A previous animal study has yielded promising results, indicating that PV can help improve the fatigue caused by high-intensity exercise by the regulation of serum fatigue biomarkers (such as the inhibition of the accumulation of lactate and inorganic phosphate) and muscle injury (antioxidant activity *via* decreased malondialdehyde (MDA) and increased glutathione peroxidase activities) (17). Data on safe doses were obtained from animal experiments. Animal models may not always be able to predict human reactions, which make it important to confirm the results in animal studies by conducting clinical trials (18). Therefore, clinical trials are necessary to confirm the efficacy of PV in improving fatigue in humans. Contrary to the expectations based on previous animal studies, supplements, especially in the form of juice, had no effect on fatigue in most clinical studies. Previous studies have reported that the placebo effect affects fatigue improvement (19, 20). These could be partially explained using the similarity in taste between a placebo and the supplement, especially in juice forms. Fatigue was evaluated through questionnaires, such as the Fatigue Severity Scale (FSS) (21, 22) and visual analog scale (VAS) (23), rather than hematological and physiological indicators because their assessment is subjective. Therefore, to ensure the validity of the participants’ questionnaire responses, we must verify the consistency between the participants’ responses to various questionnaires. To the best of our knowledge, there have been no studies examining the effects of PV on fatigue in humans. Therefore, we conducted this randomized, double-blind, placebo-controlled trial to evaluate the efficacy and safety of fermented PV in the juice form in improving fatigue in adults with unexplained fatigue, considering the placebo effect.

## Materials and methods

### Study design and ethical aspects

This is a single-center, randomized, double-blind, placebo-controlled trial. The trial was conducted from December 2019 to August 2020 at Pusan National University Yangsan Hospital. Randomization of the two study groups was performed using a random number table. The participants were assigned sequentially randomized numbers. These randomization codes were held by the company that manufactured the fermented PV and dummy placebo. The authors who selected the study participants and performed the measurements were unaware of the randomization assignments. After baseline assessment, the participants were randomly allocated to either the PV-supplemented group (PV group, *n* = 40) or the placebo-supplemented group (placebo group, *n* = 40). The participants were asked to complete their medication records. Adherence rates of ≥80% were required for optimal therapeutic efficacy. After treatment initiation, each participant was instructed to visit the clinic at 4 (± 5 days) and 8 (± 5 days) weeks. Blood pressure (BP) measurements, questionnaires, and blood tests were performed for every participant on each visit.

The study was approved by the Institutional Review Board of Pusan National University Yangsan Hospital (02-2019-038) and registered with ClinicalTrials.gov (Identifier: NCT04319692). This study was conducted in accordance with the principles of the Declaration of Helsinki. Before the commencement of the study, written informed consent was obtained from all the participants.

### Study participants

Participants aged ≥19 years who had been diagnosed with unexplained fatigue for at least 1 month were enrolled in the study. Participants with any of the following were excluded from the study: chronic hepatitis B or C; hypothyroidism or hyperthyroidism; abnormal liver or renal function (liver or renal function test levels more than two times the normal upper limit of the central laboratory [GCCL, Yongin, Republic of Korea]); uncontrolled diabetes (fasting glucose concentration >160 mg/dL); uncontrolled hypertension (BP >160/100 mmHg); notable coronary artery disease or heart failure; malignancy within the past 5 years; a history of medication for a psychiatric illness or drug intoxication; alcohol abuse; known allergies; use of any medications within the past 1 month that may affect fatigue (such as herbs, liver supplements, beta-blockers, steroids, or hormones); those who participated in other drug clinical trials within past 1 month; severe gastrointestinal symptoms; those who are pregnant, lactating, or planned to become pregnant during the clinical trial.

### Test product and placebo

Based on the results of a previous animal study (17), the final dose of 70 mL/day of fermented PV was selected for humans. The composition of fermented PV was analyzed for this clinical trial. Each juice contained 2.56 mg/g chlorogenic acid and 15.3 mg/g citric acid as the primary bioactive components (Dong-A University, Busan, Republic of Korea), and each placebo contained purified water (84.0%), sugar (12.0%), plum flavor (2.0%), vinegar flavor (2.0%), and yellow color additives (0.004%). Participants in the PV group were administered 70 mL of fermented PV (2.56 mg/g of chlorogenic acid and 15.3 mg/g of citric acid), daily for 8 weeks. Simultaneously, participants in the placebo group were administered the same quantity of placebo for 8 weeks. The test product and placebo were identical in their external forms and properties, including the label. To maintain the double blindness, test products and placebo were made so that they could not be visually distinguished. The details of the assignment of the research subject code were managed in a sealed state by the principal investigator and were not disclosed until the end of the trial. Even if reading the code was unavoidable due to the occurrence of a serious adverse drug reaction, it was managed in the form of a separate blindfolded envelope for each participant, so that only the random assignment details of the study participants with who experienced adverse reactions could be read.

### Measurements of efficacy

The primary outcome measure was the change in the FSS score within the 8-week treatment period. The scale contains nine items that measure the severity of fatigue symptoms in study participants during the past week (24). The secondary outcome measures were the changes in the fatigue VAS (25), the Korean version of the Beck Depression Inventory (BDI) second edition (26), the Korean version of the Brief Encounter Psychosocial Instrument (BEPSI-K) (27), Euro-QoL-5D (EQ-5D)-3L and EQ-VAS (28, 29), lactate, MDA, and creatine kinase (CK) concentrations within the 8-week treatment period. Additionally, VAS was assessed by asking the participant to specify their overall discomfort level by indicating a position along a continuous 100-mm line between two endpoints. The left end indicated “worst,” whereas the right end indicated “best,” and the value was subsequently determined by measuring the length (mm) from the left end of the line. The questionnaires were administered by a well-trained research assistant.

### Safety evaluation

To evaluate the safety of PV, participants were assessed for BP, pulse rate, and laboratory test results, including complete blood cell count, liver enzyme, fasting glucose, and creatinine.

### Blood chemistry

All laboratory analyses were conducted by a central laboratory. Peripheral blood, after a 12-hour overnight fast, was collected in ethylenediaminetetraacetic acid tubes at baseline and 8 weeks after the randomization, to evaluate the effectiveness and monitor for the potential adverse effects of PV. For evaluating the efficacy of PV, the CK was analyzed by the CK NAC-activated procedure using an AU5800 chemistry analyzer (Beckman Coulter, Brea, CA, USA). Lactate concentrations were measured by ion-selective electrode assay using a Stat Profile pHOx Ultra analyzer (Nova Biomedical, Waltham, MA, USA), which had an inter-and intra-assay CV of 3.0% and 5.0%, respectively. MDA concentration was assessed using the OxiSelectTM thiobarbituric acid reactive substances assay kit (Cell Biolabs Inc., San Diego, CA), which had an inter-and intra-assay CV of 2.0 and 2.0%, respectively. The relative fluorescence intensity of cells was estimated using the Varioskan Flash spectral scanning multimode reader (Thermo Fisher Scientific Inc., Waltham, MA). To monitor the potential adverse effects of PV, serum creatinine was measured using modified Jaffe’s kinetic alkaline picrate method, glucose was measured using a glucose oxidase test method (LX-20; Beckman Coulter, Fullerton, CA, USA), and liver enzyme concentrations were measured according to an enzymatic colorimetric method using an AU5800 chemistry analyzer (Beckman Coulter, Brea, CA, USA).

### Statistical analyses

The sample size was calculated using nQuery Advisor software v. 7.0 (Statsols, Cork, Ireland). The difference in pre-and post-treatment changes in the total score of the FSS between the PV and placebo groups was set at 0.64 based on a previous similar study (30). The estimated sample size was 32 participants per group for 80% power to detect a difference of 0.64, assuming a standard deviation of 0.9 in the primary outcome variable and an alpha error of 5%. Subsequently, the sample size was adjusted to 40 participants per group to allow for 20% dropouts. The data were represented using the proper characteristics, for instance, median, mean, and percentage. The primary analysis for comparing outcomes among groups with multiple imputation of missing data was intent to treat. The per-protocol analysis was also performed to assess the supplementation effectiveness. The categorical and continuous variables were analyzed using the chi-square test, the Mann–Whitney’s *U* test, or the two-sample *t*-test. Analysis of covariance (ANCOVA) or rank ANCOVA was used for the main analysis, with adjustment for age, sex, and baseline variables as covariates. Model assumptions were observed using histograms, normal probability plots, and residual scatter plots. The change from baseline to 8 weeks in outcomes was expressed as the LSmean percentage of the baseline levels using an ANCOVA model. When *P* < 0.05, the efficacy was seen to be statistically significant. Using Microsoft Excel 365 (v. 16.45 for Mac, Microsoft), the data were recorded. The analysis of all the data was carried out using SPSS v. 22.0 (IBM Inc., Armonk, NY, USA).

## Results

### Consolidated standards of reporting trials (CONSORT) flow diagram and baseline characteristics of the participants

**FIGURE 1:**
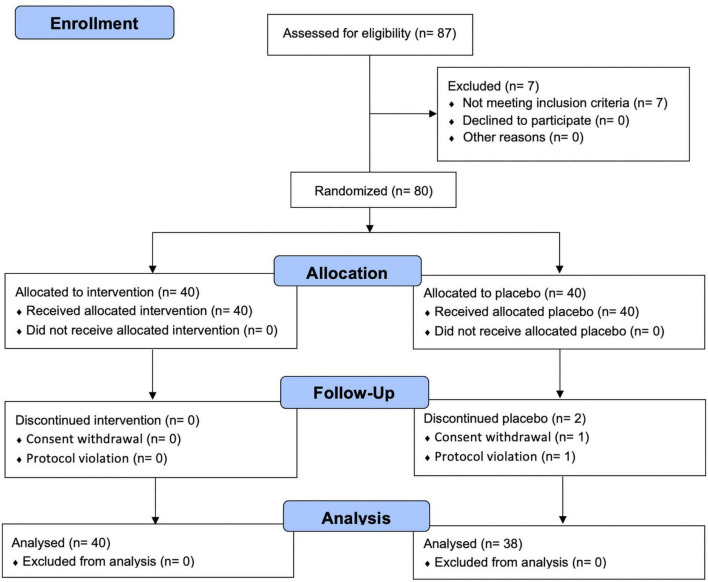
CONSORT flow diagram.

The flow of participants through the controlled interventional trial is depicted in a CONSORT conform diagram (Figure 1). Of the 87 participants screened, 80 were enrolled in the study and randomly assigned to the PV (*n* = 40) or placebo group (*n* = 40). One participant withdrew from the study, and one was excluded owing to a protocol violation of non-compliance in the placebo group; this was not associated with any treatment-related adverse effects. Overall, 78 (97.5%) participants completed follow-up assessments and questionnaires over 8 weeks, comorbid disorders were observed in three (7.5%) participants (1: hypertension, 1: gout, 1: metabolic syndrome) in the PV group and one (2.5%, migraine) participant in the placebo group. Randomization was successful, and significant differences between the groups’ baseline socio-demographic or anthropometric characteristics were not observed (Table 1). During the entire study period, the double-blind requirement was well maintained.

**TABLE 1:** Baseline characteristics of the study group.

Variables	Intention to treat population			Per protocol population		
	PV group (*n* = 40)	Placebo group (*n* = 40)	P^1^	PV group (*n* = 40)	Placebo group (*n* = 38)	P^1^
Male (%)	10 (25.0)	10 (25.0)	1.000	10 (25.0)	10 (26.3)	0.894
Age (years)	33.5 [28.5-39.0]	37.5 [31.0-46.0]	0.060	33.5 [28.5-39.0]	37.5 [32.0-46.0]	0.053
BMI (kg/m^2^)	23.0 ± 5.2	23.9 ± 3.7	0.381	23.0 ± 5.2	23.5 ± 3.4	0.606
Systolic BP (mmHg)	115.6 ± 13.9	118.3 ± 13.7	0.381	115.6 ± 13.9	117.3 ± 13.5	0.565
Diastolic BP (mmHg)	74.4 ± 9.5	76.1 ± 10.9	0.454	74.4 ± 9.5	75.2 ± 10.5	0.705
Alcohol drinker (%)	8 (20.0)	11 (27.5)	0.684	8 (20.0)	10 (26.3)	0.747
Current Smoker (%)	1 (7.5)	0 (0.0)	1.000	1 (8.8)	0 (0.0)	0.740

Values are *n* (%), mean ± SD, except for age, which is median [IQR]. PV, Fermented Prunus mume vinegar; BMI, body mass index; BP, blood pressure; ^1^*P* value by two-sample *t*-test for parametric variables, Mann–Whitney’s *U* test for non-parametric variables, and chi-square test or Fisher’s exact test for categorical variables.

### Primary outcome

**FIGURE 2:**
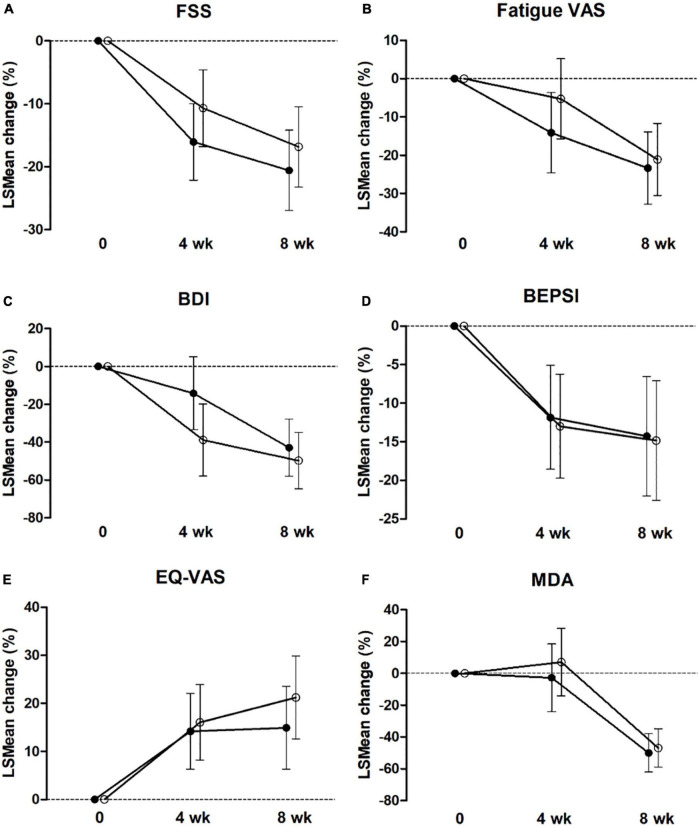
Estimated marginal mean (LS mean) change from baseline to 4 and 8 weeks for FSS **(A)**, Fatigue VAS **(B)**, BDI **(C)**, BEPSI-K **(D)**, EQ-VAS **(E)**, and MDA **(F)** in the placebo group (○) and PV group (•) by intent-to-treat analysis. Values are LSmean ± 95% CI. FSS, Fatigue Severity Scale; VAS, visual analog scale; BDI, the Beck Depression Inventory, BEISI-K, the Korean version of the Brief Encounter Psychosocial Instrument; EQ-VAS, Euro-QoL-VAS; MDA, malondialdehyde.

At 4 and 8 weeks, FSS was significantly decreased compared with that at the baseline in both groups (both, *P* < 0.001), based on ITT and PP analyses (Figure 2 and Tables 2, 3). Figure 2 shows the LSmean percent change in FSS from baseline to weeks 4 and 8 owing to the separate analysis within each group. In both groups, FSS significantly improved after 8 weeks. However, Tables 2, 3 show no intergroup difference in FSS after 4 and 8 weeks of treatment in ITT and PP analyses.

**TABLE 2:** Primary and secondary outcomes (intention-to-treat population)^1^.

	PV group (*n* = 40)			Placebo group (*n* = 40)			Adjusted difference of PV vs. placebo	
			
	Baseline	4 weeks	8 weeks	Baseline	4 weeks	8 weeks	Δ 4 weeks^2^	Δ 8 weeks^2^
Fatigue Severity Scale	4.8 ± 0.8	4.0 ± 1.0**	3.8 ± 1.0**	4.9 ± 0.9	4.3 ± 0.9**	4.0 ± 1.0**	0.20 (−0.19, 0.59)	0.13 (−0.28, 0.55)
Fatigue VAS	68.4 ± 14.1	57.5 ± 19.3**	51.1 ± 19.8**	69.1 ± 16.1	59.2 ± 15.5*	51.3 ± 19.3**	0.74 (−7.15, 8.63)	−0.46 (−9.36, 8.43)
BDI	9.0 [6.3−15.5]	7.0 [4.0−10.8]**	4.0 [1.0-8.8]**	13.5 [7.3-18.8]	9.0 [3.3-13.0]**	5.5 [1.3-9.8]**	−1.06 (−2.88, 0.76)	−0.46 (−2.99, 2.08)
BEPSI-K	2.0 [1.6-2.4]	1.6 [1.4-2.0]*	1.6 [1.4-2.0]**	2.2 [1.7-2.6]	1.8 [1.5-2.2]**	1.7 [1.4-2.0]**	−0.03 (−0.24, 0.19)	−0.07 (−0.32, 0.18)
EQ-5D-3L	0.9 ± 0.1	1.0 ± 0.1	1.0 ± 0.1*	0.9 ± 0.1	0.9 ± 0.1*	0.9 ± 0.2	0.001 (−0.03, 0.03)	−0.01 (−0.05, 0.03)
EQ VAS	63.7 ± 15.9	70.4 ± 13.6**	71.8 ± 17.2**	64.4 ± 16.4	69.2 ± 15.0	72.9 ± 14.2**	−0.52 (−6.51, 5.46)	1.75 (−4.48, 7.99)
Lactate, mmol/L	1.0 ± 0.3	1.1 ± 0.5	1.1 ± 0.4	1.2 ± 0.4	1.0 ± 0.4	1.1 ± 0.4	−0.14 (−0.32, 0.04)	−0.07 (−0.22, 0.08)
Creatine kinase, U/L	86.5 [64.3-114.0]	89.5 [65.3-126.0]	80.5 [68.3-142.3]	83.5 [65.5-144.3]	79.0 [61.3-145.8]	87.5 [64.3-164.3]	−84.68 (−227.42, 58.05)	−4.03 (−118.02, 109.96)
Malondialdehyde, μmol/L	4.6 ± 1.7	3.9 ± 1.7*	2.0 ± 1.0**	4.8 ± 1.8	4.2 ± 1.3	2.2 ± 1.0**	0.13 (−0.50, 0.77)	0.18 (−0.27, 0.63)

^1^Values are mean ± SD, median (IQR) or mean (95% CI). PV, Fermented Prunus mume vinegar; VAS, visual analog scale; BDI, Beck depression inventory; BEPSI-K, the Brief Encounter Psychological Instrument-Korean version; EQ-5D-3L, EuroQol group 5 Dimensions 3 level.

^2^ANCOVA or rank ANCOVA with adjustment for age and each baseline value as covariates over the 4-week and 8-week period (all *P* > 0.05).

*P < 0.05, **P ≤ 0.005 by paired *t*-test or Wilcoxon signed-rank test within each group.

**TABLE 3:** Primary and secondary outcomes (per protocol population)^1^.

	PV group (*n* = 40)			Placebo group (*n* = 38)			Adjusted difference of PV vs. placebo	
			
	Baseline	4 weeks	8 weeks	Baseline	4 weeks	8 weeks	Δ 4 weeks^2^	Δ 8 weeks^2^
Fatigue severity scale	4.8 ± 0.8	4.0 ± 1.0**	3.8 ± 1.0**	4.8 ± 0.9	4.2 ± 0.9**	3.9 ± 0.9**	0.19 (−0.20, 0.59)	0.07 (−0.34, 0.48)
Fatigue VAS	68.4 ± 14.1	57.5 ± 19.3**	51.1 ± 19.8**	68.4 ± 16.1	59.1 ± 15.9*	49.6 ± 18.3**	0.89 (−7.22, 9.00)	−2.05 (−10.92, 6.82)
BDI	9.0 [6.3-15.5]	7.0 [4.0-10.8]**	4.0 [1.0-8.8]**	13.0 [6.8-18.3]	9.0 [3.0-13.0]**	5.0 [1.0-9.0]**	−1.11 (−2.97, 0.76)	0.99 (−3.40, 1.42)
BEPSI-K	2.0 [1.6-2.4]	1.6 [1.4-2.0]*	1.6 [1.4-2.0]**	2.1 [1.6-2.6]	1.8 [1.4-2.1]**	1.6 [1.4-2.0]**	−0.01 (−0.23, 0.20)	−0.12 (−0.36, 0.12)
EQ-5D-3L	0.9 ± 0.1	1.0 ± 0.1	1.0 ± 0.1*	0.9 ± 0.1	0.9 ± 0.1*	0.9 ± 0.1	0.000 (−0.03, 0.03)	−0.003 (−0.04, 0.03)
EQ VAS	63.7 ± 15.9	70.4 ± 13.6**	71.8 ± 17.2**	66.0 ± 15.0	69.7 ± 14.6	75.1 ± 10.8**	−0.75 (−6.81, 5.32)	3.27 (−2.82, 9.36)
Lactate, mmol/L	1.0 ± 0.3	1.1 ± 0.5	1.1 ± 0.4	1.2 ± 0.4	1.1 ± 0.4	1.1 ± 0.4	−0.15 (−0.33, 0.04)	−0.08 (−0.23, 0.08)
Creatine kinase, U/L	83.5 [64.3-114.0]	89.5 [65.3-126.0]	80.5 [68.25-142.25]	79.5 [64.25-151.0]	79.0 [60.75-129.25]	86.0 [63.8-172.8]	−85.65 (−232.49, 61.19)	−1.17 (−118.4, 116.1)
Malondialdehyde, μmol/L	1.8 ± 1.8	4.1 ± 1.3*	2.0 ± 0.7**	4.8 ± 1.8	4.1 ± 1.3	2.0 ± 0.7**	0.09 (−0.56, 0.73)	0.001 (−0.39, 0.39)

^1^Values are mean ± SD, median (IQR) or mean (95% CI). PV, Fermented Prunus mume vinegar; VAS, visual analog scale; BDI, Beck depression inventory; BEPSI-K, the Brief Encounter Psychological Instrument-Korean version; EQ-5D-3L, EuroQol group 5 Dimensions 3 level.

^2^ANCOVA or rank ANCOVA with adjustment for age and each baseline value as covariates over the 4-week and 8-week period (all *P* > 0.05).

*P < 0.05, **P ≤ 0.005 by paired *t*-test or Wilcoxon signed-rank test within each group.

### Secondary outcome

Among the secondary outcome variables in ITT analysis, at 4 weeks, fatigue VAS, BDI, and BEPS-K were decreased compared with that at baseline in both groups (PV group; *P* = 0.001, *P* < 0.001, *P* = 0.006, respectively; placebo group; *P* = 0.006, *P* < 0.001, *P* < 0.001, respectively). Alternatively, EQ-VAS increased, and MDA increased significantly only in the PV group (*P* = 0.005 and *P* = 0.047, respectively), whereas EQ-5D-3L increased only in the placebo group (*P* = 0.011) (Figure 2 and Tables 2, 3).

These results followed the same pattern in the PP analysis (Figure 2 and Tables 2, 3). At 8 weeks, fatigue VAS, BDI, BEPSI-K, and MDA decreased, whereas EQ-VAS increased in both groups (PV group; *P* < 0.001, *P* < 0.001, *P* = 0.002, *P* < 0.001, and *P* = 0.001, respectively; placebo group; *P* < 0.001, *P* < 0.001, *P* < 0.001, *P* < 0.001, and *P* = 0.003, respectively). Additionally, EQ-5D-3L increased only in the PV group (*P* = 0.02), based on ITT and PP analyses (Figure 2 and Tables 2, 3). Figure 2 shows the LSmean percentage change in fatigue indices (fatigue VAS, BDI, BEPSI-K, EQ-VAS, and MDA) from baseline to 4 and 8 weeks as a result of a separate analysis within each group. In the PV and placebo groups, fatigue indices significantly improved after 8 weeks. However, all secondary outcomes including fatigue VAS, BDI, BEPSI-K, EQ-5D-3L, EQ-VAS, lactate, CK, and MDA concentrations did not differ between the two groups throughout the study period, based on ITT and PP analyses (Tables 2, 3).

### Correlation analysis

As shown in Table 4, partial correlation analysis after controlling for age showed that FSS had a positively correlation with fatigue VAS, BDI, and BEPSI-K, but a negatively correlation with EQ-5D-3L and EQ-VAS, based on the ITT population at baseline and 8 weeks. This trend was the same one that was observed in the PP analysis.

**TABLE 4:** Pearson’s correlation coefficient and partial correlation between fatigue severity scale and other fatigue indices.

Variables	Baseline					8 weeks				
	Values	*r*	P	*r*’	P’	Values	*r*	P	*r*’	P’
**Intention to treat population (*N* = 80)**										
FSS	4.83 ± 0.85	1		1		3.87 ± 0.97	1		1	
Age (years)	36.7 ± 10.2	−0.121	0.287			36.7 ± 10.2	0.010	0.933		
Fatigue VAS	68.7 ± 15.0	0.295	0.008	0.290	0.009	51.2 ± 19.4	0.498	< 0.001	0.498	< 0.001
BDI^1^	11.5 [7.0-17.0]	0.296	0.008	0.346	0.002	5.0 [1.0-9.0]	0.578	<0.001	0.583	<0.001
BEPSI-K^1^	2.0 [1.6-2.4]	0.270	0.015	0.279	0.013	1.6 [1.4-2.0]	0.443	0.001	0.368	0.001
EQ-5D-3L	0.92 ± 0.10	−0.198	0.079	−0.231	0.040	0.94 ± 0.11	−0.467	<0.001	−0.475	<0.001
EQVAS	64.0 ± 16.1	−0.345	0.002	−0.338	0.002	72.4 ± 15.7	−0.550	<0.001	−0.550	<0.001
Lactate, mmol/L	1.1 ± 0.4	0.158	0.160	0.183	0.106	1.1 ± 0.4	−0.153	0.174	−0.153	0.177
CK, U/L^1^	83.5 [65.0-121.0]	−0.022	0.847	0.059	0.678	84.0 [66.3-146.8]	−0.079	0.484	0.077	0.501
MDA, μmol/L	4.69 ± 1.75	−0.125	0.270	−0.082	0.471	2.06 ± 0.98	0.094	0.409	0.093	0.414
**Per protocol population (*N* = 78)**										
FSS	4.81 ± 0.85	1		1		3.82 ± 0.94	1		1	
Age	36.7 ± 10.2	−0.125	0.277			36.7 ± 10.2	0.010	0.932		
Fatigue VAS	68.4 ± 15.0	0.280	0.015	0.277	0.015	50.4 ± 19.0	0.458	<0.001	0.458	<0.001
BDI^1^	11.5 [6.8-17.0]	0.275	0.035	0.338	0.003	5.0 [1.0-9.0]	0.516	<0.001	0.549	<0.001
BEPSI-K^1^	2.0 [1.6-2.4]	0.239	0.040	0.250	0.028	1.6 [1.4-2.0]	0.403	<0.001	0.282	0.013
EQ-5D-3L	0.93 ± 0.08	−0.156	0.174	−0.213	0.062	0.95 ± 0.09	−0.417	<0.001	−0.435	<0.001
EQVAS	68.8 ± 15.4	−0.315	0.005	−0.307	0.007	73.4 ± 14.5	−0.492	<0.001	−0.492	<0.001
Lactate, mmol/L	1.1 ± 0.4	0.163	0.153	0.191	0.096	1.1 ± 0.4	−0.157	0.169	−0.157	0.172
CK, U/L^1^	80.5 [64.8-123.8]	0.038	0.739	0.064	0.582	83.5 [66.0-147.0]	0.092	0.425	0.091	0.429
MDA, μmol/L	4.68 ± 1.77	−0.133	0.246	−0.088	0.445	1.98 ± 0.37	−0.069	0.551	−0.072	0.536

Values are mean ± SD, median (IQR). FSS, Fatigue Severity Scale; VAS, visual analog scale; BDI, Beck depression inventory; BEPSI-K, the Brief Encounter Psychological Instrument-Korean version; EQ-5D-3L, EuroQol group 5 Dimensions 3 level; CK, creatinine kinase; MDA, malondialdehyde. *r*, Pearson’s or Spearman’s^1^ correlation coefficient, *r*’, age-adjusted correlation coefficient.

### Safety

All participants completed the protocol without any adverse symptoms or serious adverse events. Additionally, no participants in the two groups complained of adverse effects. After 8 weeks of the trial, no significant changes in the concentrations of liver enzymes, fasting glucose, or creatinine were observed between the two groups (Table 5).

**TABLE 5:** Laboratory findings evaluating the adverse effects and the additional benefits profile^1^.

	PV group		Placebo group		Adjusted difference of PV vs. placebo over 8 weeks	P^2^
	Baseline	8 weeks	Baseline	8 weeks		
**Intention to treat (*N* = 80)**						
AST, IU/L	20.0 [17.3-23.0]	20.0 [17.0-24.0]	20.5 [18.0-27.5]	21.5 [19.0-24.0]	1.51 (−1.22, 4.24)	0.058
ALT, IU/L	15.0 [11.0-21.0]	15.0 [12.3-20.0]	17.0 [12.0-27.0]	19.0 [14.0-23.0]	1.56 (−1.87, 4.99)	0.425
Creatinine, mg/dL	0.7 ± 0.1	0.7 ± 0.1	0.7 ± 0.1	0.7 ± 0.1	−0.02 (−0.04, 0.01)	0.212
Glucose, mg/dL	75.2 ± 6.5	80.8 ± 12.7	77.9 ± 8.6	82.7 ± 11.8	0.55 (−4.74, 5.84)	0.836
**Per protocol (*N* = 78)**						
AST, IU/L	20.0 [17.3-23.0]	20.0 [17.0-24.0]	20.5 [18.0-26.5]	21.5 [18.8-24.0]	1.65 (−1.14, 4.44)	0.049
ALT, IU/L	15.0 [11.0-21.0]	15.0 [12.3-20.0]	17.0 [12.0-25.5]	18.0 [14.0-23.0]	1.59 (−1.91, 5.10)	0.470
Creatinine, mg/dL	0.7 ± 0.2	0.7 ± 0.1	0.8 ± 0.2	0.7 ± 0.1	−0.02 (−0.04, 0.01)	0.232
Glucose, mg/dL	88.1 ± 8.1	80.8 ± 12.7	91.29 ± 11.06	82.36 ± 11.95	0.22 (−5.16, 5.60)	0.935

^1^Values are mean ± SD or median (IQR) or mean (95% CI) unless otherwise indicated. PV, Fermented Prunus mume vinegar; AST, aspartate transaminase; ALT, alanine transaminase.

^2^ANCOVA or rank ANCOVA adjusted for each baseline value as covariates over the 8-week period.

## Discussion

To the best of our knowledge, this is the first randomized, placebo-controlled study to evaluate the efficacy and safety of fermented PV on fatigue improvement in adults with subjective fatigue. Fatigue can be broadly divided into peripheral and central types. The former is related to muscle function, and the latter is associated with the central nervous system’s role in activating muscles. This fatigue means that it is mainly caused by sustained physical exercise (31). Moreover, fatigue can be evaluated with subjective and objective assessments, and has been divided into subjective and objective fatigues in this conceptual framework. The reason for the different concepts of fatigue is that there is still no universally accepted definition of fatigue. To develop a more comprehensive understanding of the complex phenomenon of fatigue, it is necessary to merge subjective and objective assessments of fatigue, but this has not yet been possible. In contrast to objective fatigue, which is assessed by quantifiable measures, subjective assessments rely primarily on self-reported perceptions (32, 33). This study used self-report instruments, such as the FSS questionnaire and fatigue VAS. Therefore, the fatigue mentioned in this study is regarded as subjective fatigue (as is based on subjective assessments). We verified the consistency between the participants’ responses to various questionnaires FSS and other questionnaires, including fatigue VAS, BDI, BEPSI-K, EQ-5D-3L, and EQ-VAS. This study showed good significant correlations between FSS and other variables (fatigue VAS, BDI, BEPSI-K, EQ-5D-3L, and EQ-VAS) at baseline and 8 weeks later. These correlations indicate that the participants’ responses to the questionnaire in the two groups are reliable (34). Nevertheless, contrary to expectations, daily intake of fermented PV for 8 weeks did not show a more remarkable effect than that of placebo on FSS, fatigue VAS, BDI, BEPSI-K, EQ-5D-3L, EQ-VAS, lactate, CK, and MDA concentrations in adults with unexplained fatigue.

In East Asia and Southeast Asia, the fruit of *Prunus mume* is used in juices, as a pickle, or even as a traditional medication for fatigue (13, 14, 35). A previous animal study has shown that the administration of 7.5% PV diluted with distilled water (7 mL/kg body weight) for 4 weeks significantly improved treadmill running time as a marker of fatigue recovery in Sprague-Dawley rats, and then increased glycogen accumulation in the muscle and liver in exhausted Sprague-Dawley rats following high-intensity exercise (17). Additionally, PV supplementation increased lactate dehydrogenase and glutathione peroxidase concentrations, while reducing ammonia, inorganic phosphate, lactate, CK, and MDA concentrations (17). However, this study showed that PV intake could affect fatigue recovery, but it did not statistically exceed the placebo effect.

However, we found a placebo effect in this study. As a result of separate analyses within each group, the PV and placebo groups showed a significant improvement in the fatigue indices after 8 weeks. The placebo effect is a phenomenon in which patients experience positive results after a fake drug is administered. Previous studies have suggested that patients’ expectations, emotions, and memories may affect the outcome of treatment (36, 37). Recent studies have reported that an open-label placebo positively affected chronic low back pain, irritable bowel syndrome, and depression (37–39). Particularly, the placebo effect has been reported in many studies on fatigue (19, 20, 40, 41). For example, in a randomized controlled study of cancer survivors, open-label placebo treatment improved the fatigue severity and fatigue-related quality of life compared with those who received the usual treatment (40). However, several randomized studies examining the effects of supplements in the form of juices (such as beetroot juice and mangosteen-based juice) on fatigue have shown no improvement in fatigue indicators after the ingestion of juice or a placebo (42–44). In a randomized, double-blind study of mangosteen, mangosteen-based juice increased the time to exhaustion by 13.3% more than the control treatment. However, there was no significant difference between the two groups (44). Another randomized, double-blind study of beetroot juice stated that beetroot juice intake improved performance in the sprint exercise. Nevertheless, this was not accompanied by differences in neuromuscular fatigue as measured by the countermovement jumps test during or after exercise (42). The present study showed that PV and placebo supplements in juice form improved fatigue indices in both groups. Although the exact mechanism of this phenomenon is not known, a placebo appears to improve fatigue by acting on the central nervous system during the preparation stage of movement (41). *Prunus mume* fruits should be processed before consumption because they contain toxic cyanoglycosides, such as amygdalin (45). Therefore, it was necessary to confirm the safety of PV for liver and kidney function. In the present study, no adverse or side effects were observed on blood clinical health markers.

The present study has some limitations. Because this study was conducted at a single medical center, there is a limit to applying the study results to the entire population. Additionally, we did not control the nutritional intake that could affect fatigue. Also, because we subjectively measured fatigue through self-report questionnaires, such as the FSS and fatigue VAS, we could not analyze it as a multidimensional state that includes cognitive, physical, and psychological factors of fatigue (46). In addition, it was difficult to determine the effect on peripheral or objective fatigue because no strength or exercise tests were performed. Therefore, there were limitations in evaluating the efficacy of fermented PV on fatigue. Despite these limitations, to the best of our knowledge, this is the first clinical study to examine the efficacy of fermented PV on fatigue recovery in adults. Although the fermented PV had not been statistically significant for fatigue, this study suggested that the placebo effect of the fermented PV could also affect the improvement of fatigue. In the current study, the taste of the placebo was reproduced similarly to the actual plum extract. The participants were not able to distinguish between placebo and PV juice at all, and at the end of the study interview, almost all of the placebo groups were considered to have been assigned to the PV group. Regrettably, the effect of fermented PV did not exceed the placebo effect in adults with unexplained fatigue. Further analysis is needed using fatigue indicators with better fluctuations.

In conclusion, fermented PV supplementation did not remarkably improve fatigue indices more than placebo in adults with unexplained fatigue. Further studies should be conducted to determine the optimal dose and duration of fermented PV administration with respect to the improvement of fatigue in humans.

## Data availability statement

The raw data supporting the conclusions of this article will be made available by the authors, without undue reservation.

## Ethics statement

The studies involving human participants were reviewed and approved by the Institutional Review Board at Pusan National University Yangsan Hospital and ClinicalTrials.gov. The patients/participants provided their written informed consent to participate in this study.

## Author contributions

SL contributed to the conceptualization of the study and coordinated and supervised the entire project. YL and SL designed the methodology of the work, had an active role in the process of participants and data acquisition, and contributed to the validation of results. JC and SL carried out the formal analysis of the data. JC, YL, and SL worked together for data curation and wrote the work’s draft and reviewed the final document. All authors contributed to the article and approved the submitted version.
